# Human Infection with *Ehrlichia muris*–like Pathogen, United States, 2007–2013^1^

**DOI:** 10.3201/eid2110.150143

**Published:** 2015-10

**Authors:** Diep K. Hoang Johnson, Elizabeth K. Schiffman, Jeffrey P. Davis, David F. Neitzel, Lynne M. Sloan, William L. Nicholson, Thomas R. Fritsche, Christopher R. Steward, Julie A. Ray, Tracy K. Miller, Michelle A. Feist, Timothy S. Uphoff, Joni J. Franson, Amy L. Livermore, Alecia K. Deedon, Elitza S. Theel, Bobbi S. Pritt

**Affiliations:** Wisconsin Department of Health Services, Madison, Wisconsin, USA (D.K. Hoang Johnson, J.P. Davis, C.R. Steward, A.K. Deedon);; Minnesota Department of Health, St. Paul, Minnesota, USA (E.K. Schiffman, D.F. Neitzel, J.A. Ray);; Mayo Clinic, Rochester, Minnesota, USA (L.M. Sloan, E.S. Theel, B.S. Pritt);; Centers for Disease Control and Prevention, Atlanta, Georgia, USA (W.L. Nicholson);; Marshfield Clinic, Marshfield, Wisconsin, USA (T.R. Fritsche, T.S. Uphoff);; North Dakota Department of Health, Bismarck, North Dakota, USA (T.K. Miller, M.A. Feist);; Mayo Clinic Health System, Eau Claire, Wisconsin, USA (J.J. Franson);; Mayo Medical Laboratories, Andover, Massachusetts, USA (A.L. Livermore).

**Keywords:** *Ehrlichia muris*, ehrlichiosis, *Ixodes scapularis*, bacteria, United States, epidemiology, vector-borne infections, zoonoses, United States, tickborne infections, surveillance, bacteria

## Abstract

An *Ehrlichia muris–*like (EML) pathogen was detected among 4 patients in Minnesota and Wisconsin during 2009. We characterized additional cases clinically and epidemiologically. During 2004–2013, blood samples from 75,077 patients from all 50 United States were tested by PCR from the *groEL* gene for *Ehrlichia* spp. and *Anaplasma phagocytophilum*. During 2007–2013, samples from 69 (0.1%) patients were positive for the EML pathogen; patients were from 5 states: Indiana (1), Michigan (1), Minnesota (33), North Dakota (3), and Wisconsin (31). Most (64%) patients were male; median age was 63 (range 15–94) years; and all 69 patients reported likely tick exposure in Minnesota or Wisconsin. Fever, malaise, thrombocytopenia, and lymphopenia were the most common symptoms. Sixteen (23%) patients were hospitalized (median 4 days); all recovered, and 96% received doxycycline. Infection with the EML pathogen should be considered for persons reporting tick exposure in Minnesota or Wisconsin.

## Abstract 2

This pathogen has been detected in patients from 5 states, all of whom reported likely tick exposure in Minnesota or Wisconsin.

Ehrlichiosis and anaplasmosis are emerging tickborne zoonoses caused by *Ehrlichia* spp. and *Anaplasma phagocytophilum*, respectively (*1*–*4*). These gram-negative obligate intracellular bacteria infect leukocytes and cause febrile illness in humans (*1*,*3*). In the United States, most infections occur during May–August, months with peak human exposure to hard tick vectors (*3*). The signs, symptoms, and clinical course of ehrlichiosis and anaplasmosis are similar; >50% of patients have fever, headache, chills, malaise, myalgia, and nausea, the most common signs and symptoms (*5*). Vomiting, diarrhea, cough, arthralgia, and confusion are less frequently reported (*3*,*6*). Rashes are infrequently reported among patients with anaplasmosis, but ≈60% of children and 30% of adults with ehrlichiosis report rash (*7*). Leukopenia, thrombocytopenia, and elevated alanine aminotransferase, aspartate aminotransferase, or alkaline phosphatase are common laboratory findings (*3*). Severe complications of anaplasmosis and ehrlichiosis are rare and may include renal failure, pneumonia, acute respiratory distress syndrome, neurologic disorder, and intravascular coagulation (*8*,*9*). Hospitalization rates are high (ehrlichiosis 49% and anaplasmosis 36%) (*1*); fatality rates are <1% among patients with anaplasmosis and 1.8% among patients with ehrlichiosis (*7*,*10*). Elderly and immunocompromised patients, including those with HIV infection and those receiving immunosuppressive therapy for malignancies, are at greatest risk for severe disease (*3*).

Anaplasmosis is reported primarily in temperate regions of North America, Europe, and Asia (*8*,*9*). In the United States, *A. phagocytophilum* infects granulocytes and is primarily transmitted by the bite of infected *Ixodes scapularis* and *I. pacificus* ticks. Anaplasmosis occurs in regions where these vectors are prevalent, principally the Northeast and upper Midwest but also Mid-Atlantic and north-central states and, less frequently, the Pacific Northwest. In contrast, human ehrlichiosis is caused primarily by *E. chaffeensis*, which infects monocytes, and less often by *E. ewingii*, which infects granulocytes. Both *Ehrlichia* spp. are transmitted through the bite of an infected *Amblyomma americanum* tick and are found in regions where this vector is prevalent, primarily the Southeast and south-central and Mid-Atlantic states. These 2 *Ehrlichia* spp. were thought to be the only causes of ehrlichiosis in the United States until 2009, when an *Ehrlichia* sp. closely related to *E. muris* was detected in 3 symptomatic patients in Wisconsin and 1 in Minnesota (*11*). This *E. muris*–like (EML) pathogen was also detected among *I. scapularis* ticks in Minnesota and Wisconsin, suggesting that this species may be a potential vector (*11*,*12*; Minnesota Department of Health, University of Wisconsin-Madison Department of Entomology, unpub. data). Testing for ehrlichiosis was not routine in these 2 states because the illness was not thought to be endemic. However, increased recognition and testing for the EML pathogen during 2009–2013 and retrospective review of PCR records during 2004–2008 resulted in identification of 69 cases, including the initially reported 4 cases (*11*). We report the clinical and epidemiologic features of EML human infections detected during 2004–2013 in the United States.

## Methods

### Real-Time PCR Testing

During January 1, 2004–December 31, 2013, real-time PCR testing was performed on DNA extracted from EDTA whole blood samples for *A. phagocytophilum, E. chaffeensis, E. ewingii*, and the EML pathogen by using a modified version of a multiplex PCR assay targeting a conserved region of the *groEL* heat-shock protein operon (*13*) (Technical Appendix Table 1). The reaction mix was prepared by using the LC FastStart DNA Master Hybridization Probes Kit (Roche, Indianapolis, IN, USA) with the following final concentrations of reagents: 3 mmol/L MgCl2, 0.5 µM each of the primers, 0.2 mmol/L each of fluorescein-labeled probes and 0.4 mmol/L of LC460-labeled probe. Amplification parameters that used the LightCycler 2.0 thermocycler (Roche) were denatured at 95°C for 10 min followed by 45 cycles of 95°C for 10 s at 20°C/s slope, 55°C for 15 s at 20°C/s slope, and 72°C for 15 s at 20°C/s slope. Melting curve analysis was performed at 95°C for 0 s at 20°C/s slope, 40°C for 60 s at 20°C/s slope, and 85°C for 0 s at 0.2°C/s slope with continuous fluorescence acquisition. Differentiation between organisms was determined by melting temperature analysis, with the EML pathogen producing results in the range of 51.5°C–53.5°C.

For available samples that tested positive by PCR for the EML pathogen during 2009–2013, confirmatory sequence analysis was performed by using a 3730 DNA Analyzer (Applied Biosystems, Foster City, CA, USA) and analyzed by using Sequencher DNA sequence analysis software version 4.2 (Gene Codes Corporation, Ann Arbor, MI, USA). PCR melting temperature data from LightCycler computer records before 2009 were also retrospectively reviewed to identify cases of EML pathogen infection.

PCR tests were performed at Mayo Medical Laboratories (MML), (Rochester, MN, USA) during 2004–2013, at MML New England (Andover, MA, USA) during 2010–2013, at the Mayo Clinic Health System (Eau Claire, WI, USA) during 2009–2013, and at Marshfield Clinic Laboratories (Marshfield, WI, USA) beginning in 2013. MML is an international reference laboratory with *Ehrlichia* and *Anaplasma* PCR testing sites in Minnesota and Massachusetts, which receive samples from all 50 United States. The Mayo Clinic Eau Claire and Marshfield Clinic sites test samples primarily from Wisconsin residents.

### Acute Phase Serologic Testing and Peripheral Blood Film Examination

Patient serum samples were tested for IgG-class antibodies reacting to *A. phagocytophilum* and *E. chaffeensis* at the Mayo Clinic, as previously described (*11*). Test results with reciprocal titers >64 were considered positive. Acute-phase samples were defined as those collected within 10 days of illness onset, and convalescent-phase samples were defined as samples collected >10 days after symptom onset. Conventional thin blood films created from selected patient samples were stained with Wright-Giemsa and examined for evidence of intraleukocytic morulae.

### Patient Information

Demographic, clinical, and epidemiologic information was obtained from health care providers of patients whose samples tested positive by PCR for the EML pathogen; standardized case report forms from the Minnesota and Wisconsin health departments were used to collect data. State health departments obtained and reviewed medical records for hospitalized patients or those treated in an emergency department. Patients were also interviewed by local and state health department staff, who used an investigation questionnaire to collect additional clinical information and travel and tick exposure history.

## Results

### Real-Time PCR Testing and Sequence Analysis

During 2004–2013, of 75,077 patient samples tested by MML (n = 63,185), Mayo Clinic Health System-Eau Claire (n = 5,722), and Marshfield Clinic Laboratories (n = 6,170), blood samples from 69 patients tested positive for the EML pathogen, including 4 previously reported patients (*11*). Sequence analysis of the *groEL* gene was performed for 64 available samples, and results showed that the amplified regions had 100% homology to each other and 98% homology to *E. muris*, thus confirming identification as the EML pathogen.

**Figure 1:**
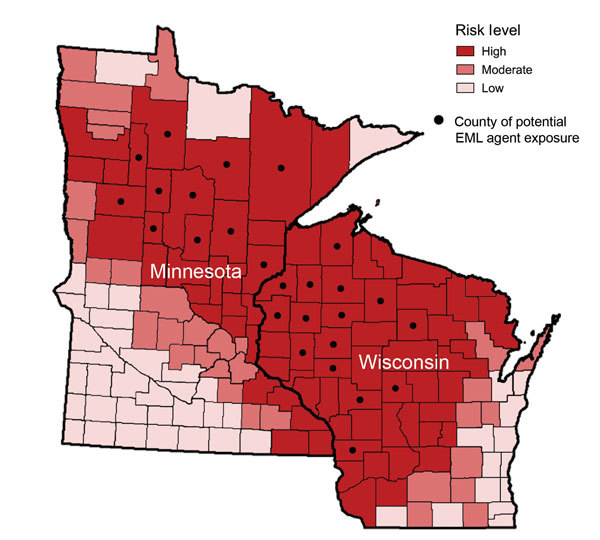
Geographic distribution of the likely county in Minnesota or Wisconsin in which exposure to the *Ehrlichia muris*–like (EML) pathogen occurred in relation to the risk for Lyme disease, babesiosis, and anaplasmosis. The risk of tickborne disease is based on county-specific mean annual reported incidence of confirmed Lyme disease and confirmed and probable human anaplasmosis and babesiosis cases in Minnesota and Wisconsin during 2007–2013. Counties with ≤10 cases/100,000 population were classified as low risk; counties with 10–24.9 cases/100,000 population were classified as moderate risk; and counties with >25 cases/100,000 population were classified as high risk.

Of the 69 positive results, 64 (93%) were detected among 39,981 samples submitted from health care providers in Minnesota (33 positive results) and Wisconsin (31 positive results) (Figure 1). Another 5 positive results (3 from North Dakota, 1 from Indiana, and 1 from Michigan) were detected among 35,096 samples submitted from the other 48 states. All 69 patients with positive results were tested during 2007–2013 and reported likely tick exposures in Minnesota or Wisconsin. No cases were detected before 2007.

Among serum samples submitted for *Ehrlichia* and *Anaplasma* PCR, 16,805 were submitted by providers in New England and Mid-Atlantic states that have high reported rates of human anaplasmosis (>3.0 cases/1 million persons annually for each state of Connecticut, Massachusetts, Maine, Rhode Island, and New York) (*1*). However, the EML pathogen was not detected in samples from these states.

After the EML pathogen was first recognized in the laboratory and alerts were issued to physicians by the Minnesota and Wisconsin state health laboratories, detection of the pathogen among Minnesota and Wisconsin residents was 7–23 (0.1%–0.3%) positive samples per year during 2010–2013 (Table 1). Retrospective review of computer PCR records identified 3 more cases from 2007–2008 and 1 more case from 2009 (not included in the initial publication) (*11*); these cases were identified on the basis of a melting temperature in the EML pathogen range. Archived samples were not available for these 4 patients; therefore, additional laboratory testing was not possible. The EML pathogen was rarely detected compared with detection of *A. phagocytophilum*. During 2013, only 17 (0.12%) of 13,639 samples from Minnesota and Wisconsin were positive for the EML pathogen, compared with 503 (3.7%) samples that were positive for *A. phagocytophilum*.

**Table 1:** Number of real-time PCR tests to detect *Ehrlichia* species*/Anaplasma phagocytophilum* performed by year and detection of the EML pathogen among samples from residents of Minnesota and Wisconsin, 2004–2013*

Testing	2004†	2005	2006	2007	2008	2009	2010	2011	2012	2013
No tests performed	275	520	779	1,224	1,909	2,198	4,365	7,349	7,723	13,639
No. (%) EML positive	0 (0)	0 (0)	0 (0)	2 (0.2)	1 (0.1)	5 (0.2)	9 (0.2)	23 (0.3)	7 (0.1)	17 (0.1)
*Numbers of patients with EML-positive samples do not include 5 EML-positive samples submitted from patients outside MN and WI (2009–2013) who likely acquired infection in MN and WI. EML, *Ehrlichia muris*–like; †First year that PCR assay was used for clinical testing.										

### Acute Phase Serologic Testing and Peripheral Blood Film Examination

Of the 69 patients with samples testing positive by PCR for the EML pathogen, samples from 6 were tested at a commercial laboratory for IgG-class antibodies to *E. chaffeensis* and *A. phagocytophilum* (Table 2; Technical Appendix Table 2). For 1 patient, an acute sample collected 6 days after illness onset (reciprocal titer 512) and a convalescent sample collected 56 days after illness onset (reciprocal titer 1,024) tested positive for IgG-class antibodies to *E. chaffeensis*. For another patient, an acute sample collected 2 days after illness onset (reciprocal titer 64) tested positive for IgG-class antibodies to *A. phagocytophilum* (Technical Appendix Table 2). For each of 15 patients whose samples tested positive by PCR for the EML pathogen, 2 thin blood films were examined by using light microscopy with oil immersion. No intraleukocytic morulae were identified in these films.

**Table 2:** Clinical features and laboratory findings among patients infected with the EML pathogen, United States, 2007–2013*

Clinical features	Value
Patient age at illness onset, y	
Range	15–94
Mean	60.8
Median	62
Sex†	
M	44/69 (64%)
F	25/69 (36%)
Period from symptom onset to testing, d	
Range	0–145
Mean	9.5
Median	4
Immunocompromised state†	13/49 (27%)
Solid organ allograft recipient	7
Receipt of chemotherapy for malignancy	2
Receipt of systemic steroids for autoimmune disease‡	4
Patient symptoms†	
Fever	60/69 (87%)
Malaise/fatigue	47/62 (76%)
Headache	46/69 (67%)
Myalgia	41/68 (60%)
Nausea/vomiting	15/69 (22%)
Rash	8/69 (12%)
Laboratory findings†§	
Anemia	18/50 (36%)
Leukopenia	20/51 (39%)
Lymphopenia	17/32 (53%)
Thrombocytopenia	34/51 (67%)
Elevated AST or ALT	18/23 (78%)
*Ehrlichia chaffeensis* positive serology, acute	1/6 (17%)
*Anaplasma phagocytophilum* positive serology, acute	1/6 (17%)
*Borrelia burgdorferi* positive serology or PCR	2/28 (7%)
Doxycycline treatment†¶	66/68 (96%)
Length of treatment, d	
Range	7–30
Mean	15.0
Median	14
Hospitalization†#	16/69 (23%)
Length of stay, d	
Range	2–15
Mean	6.1
Median	4
Immunocompromised patients†	10/15 (67%)
Death†	0/69 (0%)

*ALT, alanine aminotransferase; AST, aspartate aminotransferase; EML, *Ehrlichia muris*–like. †Expressed as number of patients with a specific risk factor, symptom, laboratory finding, or outcome, divided by the number of patients with available data. Corresponding percentage is also provided. ‡Of the 4 patients receiving systemic steroids for an autoimmune condition, 3 had rheumatoid arthritis, and 1 had mixed connective tissue disease. §Anemia, hemoglobin <13.5g/dL for male or <12.0g/dL for female patients; leukopenia, <3.5 cells x 10^−9^/liter; thrombocytopenia, <150 cells × 10^−9^/liter; elevated AST, >48 U/liter; elevated ALT, >55 U/liter; positive *Ehrlichia chaffeensis* or *Anaplasma phagocytophilum* serology, reciprocal IgG titer >64. ¶Two patients recovered without treatment. Treatment information was unavailable for 1 patient. #Length of stay was unknown for 2 of the 16 hospitalized patients.

### Clinical and Epidemiologic Features

**Figure 2:**
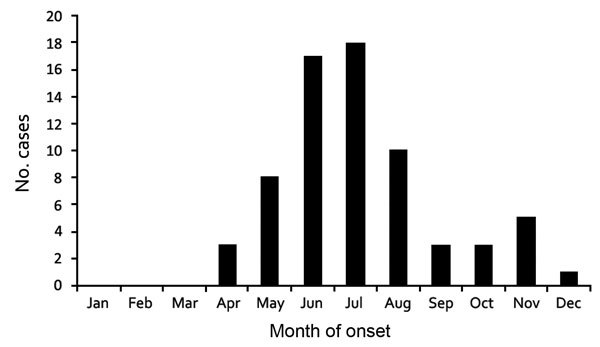
Month of symptom onset among 68 patients with *Ehrlichia muris*–like pathogen infection detected during 2007–2013, United States. Month of symptom onset was unknown for 1 patient.

Among the 69 EML-positive patients, 44 (64%) were male; age range was 15–94 (median 62) years (Table 2). Patients reported symptom onset during April–December, with peak onset (52%) occurring in June and July (Figure 2). Thirteen (27%) of 49 patients with known immune status had immunocompromised conditions resulting from immunosuppressive therapies: 7 with solid organ allografts received immune modulating pathogens; 2 received chemotherapy to treat malignancy; and 4 received systemic steroids to treat an autoimmune disease (rheumatoid arthritis [n = 3] and mixed connective tissue disease [n = 1]). The most frequently noted signs and symptoms were fever (87%), malaise (76%), headache (67%), and myalgia (60%). Frequently noted laboratory findings among those tested included thrombocytopenia (67%), lymphopenia (53%), leukopenia (39%), anemia (36%), and elevated aspartate aminotransferase or alanine transaminase (78%) (Table 2). Two of 28 patients tested had positive serologic results for *Borrelia burgdorferi*.

The range of days from symptom onset to PCR testing was 0–145 (median 4) days; 59 (86%) of 69 cases were detected within 10 days, and 94% were detected within 21 days. One patient reported new onset of fatigue and sweats ≈4 months before testing, but because of a complex medical history, a more compressed period for EML infection could not be excluded. The hospitalization rate was 23% (16/69 patients), and length of stay was 2–15 (median 4) days (Table 2). All patients recovered. Of 68 patients with available treatment information, 66 (97%) received a course of doxycycline and 2 recovered without therapy.

## Discussion

We report clinical and epidemiologic data for 69 symptomatic patients infected with the newly described EML pathogen, including 4 patients with previously reported cases (*11*). All patients reported likely exposure to ticks in Minnesota or Wisconsin, and the EML pathogen was not detected in >16,000 patient samples from other US states that have high incidence of diseases caused by other tickborne pathogens transmitted by *Ixodes* spp. ticks. The relatively limited area where EML infections were likely acquired suggests that the EML pathogen may have a limited geographic distribution. This potential geographic focus is also supported by tick data in which the EML pathogen has been detected only among *I. scapularis* ticks from Minnesota and Wisconsin and not from *I. scapularis* and *A. americanum* ticks from other US states (Minnesota Department of Health; University of Wisconsin–Madison Department of Entomology; and US Army Department of Defense Human Tick Test Kit Program, unpub. data) (*12*,*14*). Additional testing of humans and ticks from regions outside the upper midwestern United States would be valuable for further defining the distribution of the EML pathogen.

Data accumulated to date indicate that the epidemiologic features and clinical signs and symptoms of infection with the EML pathogen are similar to those of anaplasmosis and ehrlichiosis caused by other pathogens. Illness onset peaked in June and July, consistent with infection occurring during peak *I. scapularis* nymph activity of May and June in Minnesota and Wisconsin. Infected patients were more frequently male (1.8:1), and average patient age was 61 years. Fever, malaise, headache, and myalgia were the most common symptoms; leukopenia, lymphopenia, thrombocytopenia, and elevated levels of hepatic transaminase were the most common laboratory findings, consistent with anaplasmosis and ehrlichiosis caused by *E. chaffeensis* and *E. ewingii* (*1*,*3*,*15*). No intraleukocytic morulae were identified on peripheral blood films, suggesting that this method may not be sensitive enough to detect this infection. The type of leukocyte infected by the EML pathogen is unknown. 

Among 49 patients with reported immunocompromised status, 13 (27%) were receiving systemic immunosuppressive therapy. This percentage of patients with immunocompromising conditions is higher than that previously reported among patients with anaplasmosis or other causes of ehrlichiosis (*1*,*8*). Consequently, immunocompromised patients may be particularly susceptible to infection with the EML pathogen. Most (7/13) immunosuppressed patients were solid organ allograft recipients; others were receiving immunosuppressive therapies for malignancy or autoimmune conditions. Two of 28 patients for whom information was available had positive serologic tests for *B. burgdorferi*. These results highlight the possibility of co-infection with other tickborne pathogens, an unexpected occurrence if *I. scapularis* ticks are the primary vector of the EML pathogen.

Commercial serologic testing of samples from 2 patients with EML infection showed positive IgG antibody results to *E. chaffeensis* or *A. phagocytophilum*. This finding supports data from a previous study that suggests that cross-reactivity can occur between *A. phagocytophilum* and *Ehrlichia* spp. (*16*). Additional serologic studies are needed to determine how frequently cross-reactivity occurs between the EML pathogen and other *Ehrlichia* spp. This information would help assess whether current commercially available *E. chaffeensis* serologic tests are reliable for testing for evidence of EML infection.

The population tested during our study does not necessarily represent the population of infected patients but represents patients who sought medical evaluation for their illnesses and had blood submitted for *Ehrlichia* and *Anaplasma groEL* PCR testing at Mayo Clinic or Marshfield Clinic laboratories. The 75,077 samples do not necessarily represent individual patients because multiple samples could have been submitted from some patients. The MMLs began testing for the EML pathogen during 2004. The absence of detection of the EML pathogen in human samples before 2007 is likely related to the small number of tests performed during that period and may indicate a low prevalence of EML infections in humans. However, detection of *E. muris* sequences that closely resemble the EML DNA pattern in archived *I. scapularis* ticks collected in Wisconsin during the 1990s suggests that the EML pathogen had been established in Wisconsin for >2 decades (*17*).

In summary, our findings indicate that human infection caused by the EML pathogen continues to occur. This pathogen should be considered among the differential diagnoses when tickborne diseases are suspected among residents of Minnesota or Wisconsin or among persons with histories of travel to either state.

Technical Appendix Tables with additional laboratory testing information for patients infected with *Ehrlichia muris*–like Pathogen, United States, 2007–2013.
