# Supplementary material for: Human Infection with Ehrlichia muris–like Pathogen, United States, 2007–2013
Source: Emerg Infect Dis. 2015 Oct;21(10):1794–9. doi: 10.3201/eid2110.150143 (PMC4593436; doi:10.3201/eid2110.150143)
Supplement: Technical Appendix — Tables with additional laboratory testing information for patients infected with Ehrlichia muris–like Pathogen, United States, 2007–2013. [file 15-0143-Techapp-s1.pdf]

Article DOI: http://dx.doi.org/10.3201/eid2110.150143

Human Infection with the *Ehrlichia muris*–like Pathogen, United States, 2007–2013

Technical Appendix

Technical Appendix Table 1. Primers and probes used in the *gro*EL heat-shock protein operon PCR assay used for testing animal and tick specimens, United States, 2007–2013*

Name	Primer and probe sequence (5′ to 3′)†
Primer 1	TAC TCA GAG TGC TTC TCA ATG T
Primer 2	GCA TAC CAT CAG TTT TTT CAA C
Fluor probe a‡	ATT TCA GCT AAT GGA GAT AAG AAT ATA
Fluor probe b‡	CAT TGT CTG CGA ATG GAG ACA AGA ACA TAG GA
LC640 probe§	GTA AGA TTG CAC AGT GTG TTC AAG AAG TCG GTA

*These sequences represent a slightly modified version of the originally published assay (*1*).
†Primer and probe sets are available through TIB MolBiol, kit no. 126 (Adelphia, New Jersey, USA).
‡Labeled with fluorescein on 3′ end.
§Labeled with LC640 on 5′ end and a phosphate on 3′ end.

Technical Appendix Table 2. Commercially tested serology results for acute and convalescent phase serum IgG antibodies against *Anaplasma phagocytophilum* and *Ehrlichia chaffeensis*, 2007–2013*

			Reciprocal IgG titers	
Patient number	State of residence	Days from illness onset to specimen collection	*A. phagocytophilum*	*E. chaffeensis*
6†	WI	2	<64	<64
8	WI	6	<64	512
8	WI	56	<64	1024
9	MN	2	<64	<64
11	ND	3	<64	<64
13	WI	7	<64	<64
18	WI	2	64	<64
56	ND	3	<64	Not Done

*Acute phase is defined as ≤10 days after symptom onset; convalescent phase is defined as
†Complete test results for Patient 6 have been previously published as Patient 2 (*2*).

References

1. Pritt BS, Sloan LM, Hoang Johnson DK, Munderloh UG, Paskewitz SM, McElroy KM, et al. Emergence of a new pathogenic *Ehrlichia* species, Wisconsin and Minnesota, 2009. N Engl J Med. 2011;365:422–9. PubMed http://dx.doi.org/10.1056/NEJMoa1010493</jr>

2. McQuiston JH, Paddock CD, Holman RC, Childs JE. The human ehrlichioses in the United States. Emerg Infect Dis. 1999;5:635–42. PubMed http://dx.doi.org/10.3201/eid0505.990504
